# Mitigation of haemato-genotoxic and stress response effects in *Cyprinus carpio* via silymarin dietary supplementation following deltamethrin exposure

**DOI:** 10.1016/j.heliyon.2024.e28419

**Published:** 2024-03-27

**Authors:** Rajinder Jindal, Ritu Sharma, Parminder Kaur, Sukhmani Kaur, Cristiana Roberta Multisanti, Caterina Faggio

**Affiliations:** a Aquatic Biology Laboratory, Department of Zoology, Panjab University, Chandigarh, 160014, India; b Department of Hepatology, Postgraduate Institute of Medical Education and Research, Chandigarh 160014, India; c Department of Biosciences, University Institute of Biotechnology, Chandigarh University, Punjab, India; d Department of Environment Studies, Panjab University, Chandigarh, 160014, India; e Department of Veterinary Sciences, University of Messina, Viale Giovanni Palatucci snc, 98168 Messina, Italy; f Department of Chemical, Biological, Pharmaceutical and Environmental Sciences University of Messina-Italy Messina, Italy; g Department of Eco-sustainable Marine Biotechnology, Stazione Zoologica Anton Dohrn, Naples, Italy

**Keywords:** Deltamethrin, Silymarin, *Cyprinus carpio*, Haematology, Cytotoxicity

## Abstract

The study examined the potential of Silymarin, a blend of bioactive flavonolignans extracted from the milk thistle Silybum marianum, to mitigate Deltamethrin-induced toxicity in the blood of *Cyprinus carpio*. Fish were exposed to Deltamethrin (0.66 μg/L), the plant extract, or a combination of both for a duration of thirty days. Various parameters, including serum biochemical markers, erythrocytic abnormalities, and genotoxicity endpoints, were assessed. Results indicated a significant (p < 0.05) increase in the levels of AST, ALT, ALP, blood urea nitrogen, creatinine, glucose, cholesterol, and TLC in the fish exposed to the pesticide. Conversely, total protein, TEC, and Hb showed a notable decrease. There was also a notable rise in micronuclei and erythrocytic abnormalities such as acanthocytes, microcytes, and notched cells. Under ultrastructural examination, phenotypic deformities like spherocytosis, discocytes, and clumped erythrocytes were observed. However, dietary supplementation of silymarin (1 g/kg) significantly restored the biochemical, genetic, and cellular parameters, resembling those of the control group. This suggests the potential of this plant extract in protecting the common carp, *Cyprinus carpio,* from Deltamethrin-induced damage by scavenging free radicals and reducing DNA oxidative stress.

## 1 Introduction

The expansion and progress observed in the agricultural industrial sector are notably paralleled by an accompanying escalation in the application of pesticides and fertilisers [[1], [2], [3], [4], [5], [6], [7]]. About 60–70% of streams, rivers, and lakes around the world are contaminated indirectly by agricultural pesticides [8]. While their application contributes to increased food production, it is also linked to potential risks such as water body contamination and adverse health effects on non-target organisms [9,10]. Unsystematic and unabated use of these agrochemicals endangers the aquatic ecosystems in areas adjacent to agricultural fields, ultimately endangering non-target aquatic organisms [[11], [12], [13], [14], [15]]. Within this context, pesticides predominantly can be absorbed through fish gills, skin, or the digestive system, subsequently making their way to various tissues through the circulatory system [[16], [17], [18]].

Deltamethrin, chemically classified as (S)-a-cyano-3-phenoxybenzyl-(1R)-cis-3(2,2-dibromovinyl)-2,2-dimethyl cyclopropane carboxylate, stands as a widely endorsed and frequently employed substance in agriculture and forestry due to its notable attributes, including exceptional efficacy, biodegradability, and comparably low levels of toxicity to mammals [[16], [17], [18]]. Given deltamethrin's proclivity for persistence and accumulation in aquatic ecosystems, this synthetic pyrethroid has been identified as a noteworthy contributor to water contamination, infiltrating aquatic ecosystems through mechanisms such as agricultural runoff and dispersion from both aerial and ground-based pesticide applications. This introduces a considerable threat to fish populations, which are known for their diminished tolerance to pesticides. Pyrethroids, such as deltamethrin, exhibit potent neurotoxic properties [19]. Indeed, these insecticides have been found to be able to impact the sodium channel within the nervous system, resulting in a prolonged sodium tail current. In this context, the non-selective nature of insecticides makes them able to impact both target and non-target species, underscoring the lack of discrimination in their effects. Consequently, it is not unexpected that a chemical designed to influence insects' biological systems could also be able to elicit comparable effects in higher life forms [20].

As a response to mitigate the detrimental impact of xenobiotics on aquatic organisms, the exploration of additive-based treatments sourced from natural origins is progressively gaining prominence and attention [[21], [22], [23], [24], [25], [26], [27]]. The worldwide exploration of the modulatory role of antioxidants found within phytochemicals, as a means to diminish free radical-induced damage, underscores the growing significance of this area of research [13]. In this context, recognised as “milk thistle” is *Silybum marianum*, a member of the Asteraceae family, which holds a significant status as a herb with remarkable antioxidant properties [28,29]. Silymarin is often used as a dietary supplement for liver-related diseases. It is believed to possess antioxidant, anti-inflammatory, and hepatoprotective properties that help protect liver cells and promote their regeneration. The potential antioxidant activity of silymarin has been extensively studied and may help reduce oxidative stress in the body [[21], [22], [23]].

*In vitro* and *in vivo* studies have shown that silymarin protects against CCl4-induced hepatotoxicity through scavenging free radicals and reactive oxygen species [30]. In addition, it inhibits lipid peroxidation, increases cellular glutathione (GSH) content, and stimulates protein synthesis [31]. Ahmadi et al. [32] and Banaee et al. [33] reported that silymarin had positive effects on the immune system of rainbow trout (*Oncorhynchus mykiss*). Shiau et al. [34] found that silymarin decreased curcumin-induced mortality of embryos and larvae of zebrafish (*Danio rerio*). In addition, the protective effect of silymarin in preventing different xenobiotic-induced hepatotoxicity [35], cardiotoxicity [36], nephrotoxicity [36,37], and neurotoxicity [38] has been previously proven.

Silymarin has been studied in aquaculture as a potential addition to fish diets. Some studies suggest that the inclusion of silymarin in fish diets may benefit their health, growth and flesh quality. Other studies have indicated silymarin's potential to reduce oxidative stress in organs such as the liver, brain and kidneys [26]. Moreover, it has also been found to exhibit neuroprotective attributes [39], alongside demonstrating anti-cancer effects [40], and anti-diabetic properties [41]. Several studies propose that the incorporation of silymarin into the diets of fish can positively impact their health, growth, and the quality of their flesh. For instance, investigations involving Nile tilapia (*Oreochromis niloticus*) [32], turbot (*Scophthalmus maximus* L.) [42], and grass carp (*Ctenopharyngodon idella*) [43] indicate that supplementing with silymarin enhances survival rates and promotes growth performance. Indeed, fish are designed as suitable bioindicators to assess the health of aquatic ecosystems [44]. Among non-target aquatic organisms, fish are relatively more sensitive to changes in their surrounding environment, and are most affected by such chemical exposures. For instance, the common carp (*Cyprinus carpio*), stands as a highly adaptable and widely cultivated freshwater fish species. Its utilization in studies is particularly valuable in the context of evaluating the overall health of aquatic ecosystems [[45], [46], [47], [48]]. In the context of pesticide pollution, *C. carpio* specimens' exposure to these contaminants is mainly attributed to the widespread cultivation of this fish in earthen ponds located near agricultural fields [49,50]. Specifically, in the context of environmental assessment and monitoring, alterations in the haematological profile of fish serve as a pivotal early warning system, offering invaluable insights into the organism's responses to xenobiotic exposure [51,52]. Oxidative damage to RBC can alter cellular morphology, and conformation of membrane proteins, and can cause lipid peroxidation which leads to hemolysis of RBC [53]. Oxidative stress hampers the oxygen delivery to the cell, which further pushes the cell toward ageing [54]. Pyrethroids, being hydrophobic easily pass through the cell membrane and interact with DNA through its acid moiety, leading to a variety of structural modifications in DNA [55]. This approach is not only instrumental in gauging the environmental impact of foreign substances but also contributes significantly to our understanding of the ecosystem's health and resilience. Therefore, haematological parameters in various fish species are consistently utilized due to their potential as highly sensitive biomarkers, and they occupy a pivotal position within the broader landscape of environmental monitoring methodologies [56,57]. Erythrocytes are major cells in circulation and can be isolated and handled providing an excellent model for different assays [58]. Harmful substances, whether through direct interaction or indirect effects, possess the potential to instigate detrimental changes in the structural integrity of the erythrocyte cytoskeleton. This interference can extend to the disruption of ion permeability and the intricate metabolic processes within red blood cells (RBCs). The cumulative impact of these disruptions subsequently culminates in a series of morphological anomalies, underscoring the profound and multifaceted influence that toxic agents can exert on the physiology and functionality of these vital blood components [53].

There is little information on the role of medicinal plants in mitigating pyrethroid-induced toxicity in fish. *C.carpio*, a widely cultured freshwater carp. Taking into account the enormous therapeutic advantages and easy availability of silymarin*,* Given the aforementioned reasons, in the present investigation, an effort has been made to assess the ameliorating effect of silymarin against the deleterious effects of DM-induced toxicity in the blood of *C. carpio,* considering biochemical, genotoxicity and ultrastructural studies.

## 2 Materials and methods

### 2.1 Fish, acclimatization, and handling

Healthy specimens of *C. carpio* (length, 10 ± 2 cm; wt., 10 ± 2 g) were procured from a local fish farm “Ajaib 95 Singh Fish Farm, Dera Bassi Panjab (India)”. Fish were acclimated for 15 days in dechlorinated water. Continuous aeration and maintenance of ambient temperature (24 ± 2 °C) were ensured using aerator filters and heaters, and fed with commercially available pelletized fish feed, “Gold Tokyo”, manufactured by Tairopet Product Pvt. Ltd., Chennai, India.

Feed remains and excretory wastes were siphoned off regularly to avoid contaminated-related stress in fish.

### 2.2 Chemicals

The toxicant administered to *C. carpio* consisted of commercial-grade DM 2.8 EC (2.8% w/w), procured from Bayer Crop Science Ltd. in India*.* To study the modulatory effects of *Silybum marianum*, Silymarin extract (70% silymarin, Zenith Nutrition, Medizen Labs, Bangalore, India) in the form of capsules (400 mg/capsule) was used at a dose of 1 g/kg diet. Experiments were conducted according to guidelines of the Institutional Animal Ethical Committee, Panjab University, Chandigarh (PU/IAEC/S/14/151). Water chemical-physic parameters during acclimation and experimental period analysed were: temperature 22 ± 2 °C, hardness 135 ± 5 mg/L, pH 7.2 ± 0.2 and D.O. 8–10 mg/L [59,60].

### 2.3 Tests for acute and chronic toxicity

Following acclimation, a semi-static toxicity bioassay was carried out to determine 96h LC_50_ of DM to *C. carpio* by Probit analysis [61] and was 2 μg/L. In the context of the chronic toxicity bioassay, fish were subjected to a sub-lethal concentration of 0.66 μg/L (equivalent to one-third of the 96-h LC50 of DM) for a duration of 30 days. The concentration chosen is environmentally relevant and has been reported in the surface water [62]. The experiments were conducted in plastic “Syntax” tanks (640 × 325 × 3345 mm), following the standard methods [62]. Fish were randomly selected and distributed into four groups: The acclimated fish were randomly distributed into three groups in nontoxic plastic tanks (Syntax 60 cm _ 30 cm _ 30 cm) containing 30 L of water. Group 1 served as control and was maintained in pesticide-free water. The fish of group II sublethal concentrations of deltamethrin, group III served as positive control, while group IV was exposed to DM (0.66 μg/L) and supplemented with silymarin (1 g/kg diet); in a semi-static system. The experiment was performed in triplicates (6 fish/tank).

• Group I – kept in dechlorinated tap water served as control;
• Group II – exposed to DM (0.66 μg/L);
• Group III – treated with diet supplementation of silymarin (1 g/kg diet);
• Group IV – exposed to DM (0.66 μg/L) and treated with silymarin supplemented diet (1 g/kg diet).

On the 30th day (the sampling day), the peripheral blood of the fish was drawn by cardiac puncture by Inserting a needle perpendicular to the ventral surface of the fish in the centre of an imaginary line between the anterior-most part of the base of the pectoral fins. The blood was immediately processed for biochemical, haematological, genotoxic and SEM studies.

### 2.4 Serum biochemistry

Peripheral blood samples collected from fish (n = 6, each group) were coagulated for 1h at 4 °C, and centrifuged at 10,000 rpm for 10 min to separate serum and stored at −20 °C until further analysis. The enzymatic activities of ALT, AST and ALP liver biomarkers were assessed according to the method given by Henry et al. [63]. Blood urea nitrogen was determined following the method given by Chaney and Marbach [64] creatinine was estimated by modifying Jaffe's method [65]. Glucose and total protein content were determined according to the method given by Trinder [66] and Lowry et al. [67] respectively. The method developed by Röschlau et al. [68] was employed for cholesterol estimation.

### 2.5 Haematological analysis

The haemoglobin estimation is done by Sahli's method. In this method, the haemoglobin in red cells was converted into acid haematin. The brown colour so developed was matched against the standard brown-tinged glass in the comparator by direct vision. Reading was taken directly as g Hb/100 ml blood. For the total erythrocyte count (TEC) and total leucocyte count (TLC) estimated, blood collected in vials containing EDTA was used for total cell counting with the help of a haemocytometer (Neubaur's counting chamber) with an improved ruling (Helige West Germany). Using an RBC pipette, the blood was drawn up to 0.5 mark and the diluting fluid to 101 mark. Counted the no. of red blood cells in 80 small squares**.** and using a white cell pipette, the blood was drawn up to 0.5 mark and the diluting fluid to 11.0 mark. Counted the no. of white blood cells in 64 squares.

### 2.6 Genotoxicity studies

#### 2.6.1 Micronucleus assay

A thin, homogeneous layer of blood was smeared on oven-dried, clean microscopic slides, fixed in methanol for 40 min, and left to air dry at room temperature for 24 h. The slides were finally stained with 5% Giemsa (in Sorenson's buffer, pH 7.2) for 45 min. A total of 1200 erythrocytes (3 slides/fish, 3 fish/group) for each group were observed under the light microscope. Cell count and frequency were transformed into percentage values.

For scoring of MN, the cells with the following criteria were considered: First, identified as extranuclear round bodies, roughly one-third of the nucleus. Second, it should stain similarly to the main nucleus, third, it must be distinct from the main nucleus, and fourth, it must share an exact plane of focus [69].

#### 2.6.2 Cellular abnormalities analysis

The slides were also scored for erythrocyte cytoplasmic abnormalities (ECA), identified according to Anbumani and Mohankumar [70]. The ECAs include echinocytes (Ec), cells with irregular surface and numerous spine-like projections at the periphery of the cytoplasm; whereas cells having fewer projections were termed as acanthocytes (Ac). Cells with marked marginal depth or indentation into the cytoplasm were considered notched (Nc) and microcytes (Mc) with a size less than one-third of a normal erythrocyte. 1000 cells from each slide (duplicate from each fish) were randomly scored under a light microscope (Olympus Magnus MLXi) with a camera (Jenoptik Germany) at a final magnification of 1000 × at SAIF, CIL, Panjab University, Chandigarh.

### 2.7 Ultrastructural studies

For SEM method given by Simpson [71] was followed. Two drops of blood were immediately fixed in 1% buffered glutaraldehyde (1% in 0.2 M phosphate buffer, pH 7.2) for 10–15 min. Fixed blood was centrifuged at 1500 rpm for 5–10 min. Extra fixative was removed and the erythrocyte pellet was completely washed thrice with 0.1 M phosphate buffer. The pellet was then gently suspended in a buffer and a small drop of the suspended erythrocytes was placed over silver tape attached to the aluminium stub. The cells were allowed to settle, air dried and coated with gold, and viewed under a scanning electron microscope (JEOL JSM-6490, 10-20Kv) at the Department of Geology, Panjab University, and Chandigarh.

### 2.8 Statistical analysis

Statistical analysis was carried out using SPSS version 21. The data was tested for normal distribution using Kolmogorov-Smirnov test. Since the data was normally distributed, the significance of differences between the control and treated groups was tested using the one-way analysis of variance (ANOVA) followed by Tukey's post hoc test for all the parameters to assess the variations in means across the different variables. A significance level of p < 0.05 was adopted to establish statistical significance. The results were expressed in the form of mean values accompanied by their standard errors (SE) for clarity and precision.

## 3 Results

### 3.1 Biochemical alterations

Prominently, the biochemical alterations are usually the first detectable and quantifiable response to environmental changes in animal health and the internal environment of the organism and provide extensive information about fish oxygen transport capacity, immune potential, level of stress, disease, intoxication, nutritional status Measurement of serum biochemical parameters such as total protein and glucose is valuable to ascertain the toxicity impact along with the overall health status of animals [72].

**Fig. 1:**
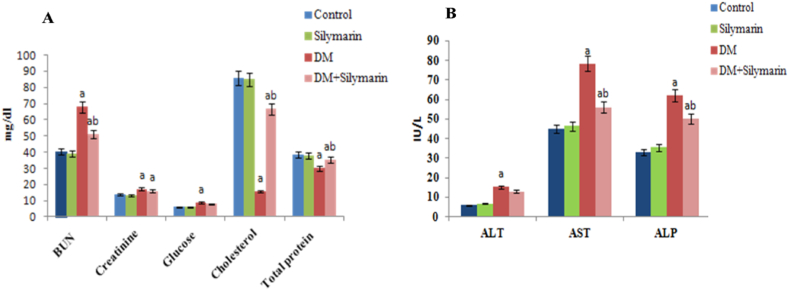
(A) Variation in serum concentration of Blood urea nitrogen (BUN), creatinine, glucose, cholesterol and total protein (mg/dl) and 1 (B) serum ALT, AST and ALP (IU/L) in *C.carpio* exposed to DM and orally supplemented with silymarin alone and in combination for 30 days. Data are presented as mean ± SD (n = 6). p < 0.05 is considered to be statistically significant, determined by one-way ANOVA followed by Tukey's post hoc test 'a’ indicates a statistically significant difference with respect to control. ‘b’: DM (0.66 μg/L) vs DM (0.66 μg/L + Silymarin).

In the present study, no significant changes in serum parameters were observed between the control group and silymarin-alone treated groups throughout the experiment. Blood urea nitrogen and creatinine levels in DM-exposed fish registered significant (p < 0.05) increased levels (68.2% and 24.60% respectively), whereas exposure to silymarin along with DM depicted a significant reduction in urea by 25.2% and creatinine by 7.4%. (Fig. 1A).

A rise in glucose and cholesterol levels by 44.7% and 814.0 % respectively, and a decrease in protein concentration by 22.7% was recorded in DM-exposed fish. Dietary administration of silymarin decreased the glucose levels declined (11.2%), and protein levels elevated (17.5%) after silymarin supplementation (Fig. 1A). The activity of ALT increased by 163.27%, AST by 73.76% and ALP by 81.8% in the DM-exposed fish groups. On the other hand, concomitant treatment of DM with silymarin showed a significant reduction in AST (28.41%), ALT (14.86%) and ALP (16.6%) activity, corresponding to DM alone treated groups after 30 days (Fig. 1B). Combined treatment of DM with silymarin resulted in a gradual recovery of altered ALP activity by 16.67%.

### 3.2 Haematological findings

**Fig. 2:**
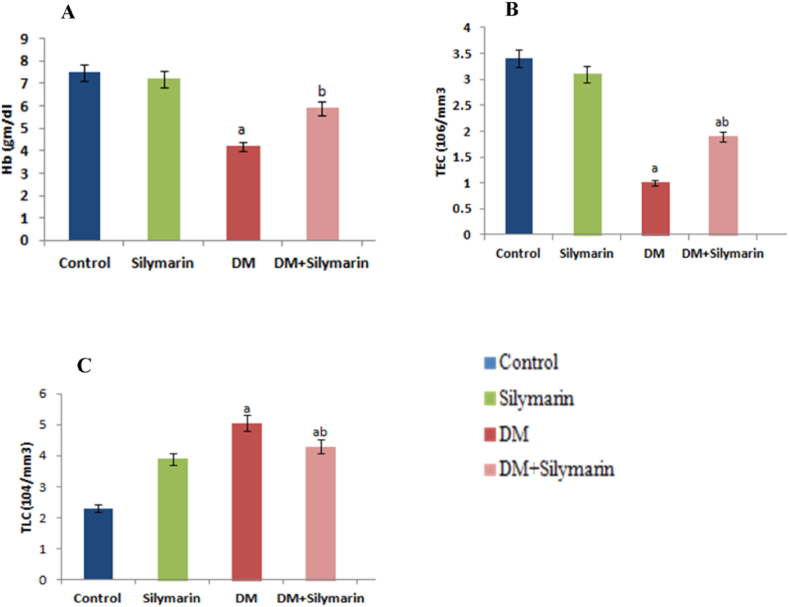
(A) Variation in Hb concentration, (B) total erythrocyte count (TEC), and (C) total leucocyte count (TLC) in *C.carpio* exposed to DM (DM) and orally supplemented with silymarin alone and in combination after 30 days. Data are presented as mean ± SD (n = 6). p < 0.05 is considered to be statistically significant, determined by one way ANOVA followed by Tukey's post hoc test 'a’ indicates a statistically significant difference with respect to control. ‘b’: DM (0.66 μg/L) vs DM (0.66 μg/L + Silymarin).

A significant (p < 0.05) reduction in Hb concentration (Fig. 2A) and total erythrocyte count (TEC) were recorded in DM-exposed *C. carpio,* while total leucocyte count (TLC) showed marked elevation in comparison to the experimental control (Fig. 2B and C). Simultaneous treatment of silymarin and DM lowered the TLC count by 14.8%, and normalized TEC and Hb content compared to DM alone treated group.

### 3.3 Micronucleus and cellular abnormalities assay

**Fig. 3:**
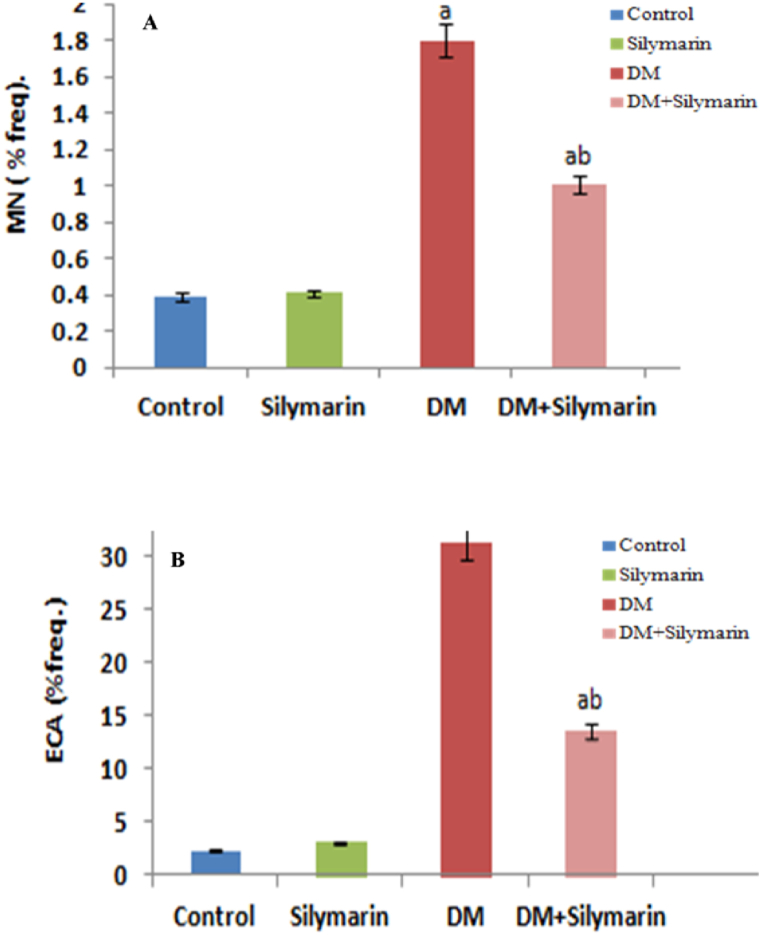
(A) Variation in Percent frequency of micronuclei and (B)ECA in the peripheral erythrocytes of *C. carpio* exposed to DM and orally supplemented with silymarin alone and in combination after 30 days. Data are presented as mean ± SD (n = 6). p < 0.05 is considered to be statistically significant, determined by one-way ANOVA followed by Tukey's post hoc. ‘a’ indicates a statistically significant difference with respect to control. ‘b’: DM (0.66 μg/L) vs DM (0.66 μg/L + Silymarin).

**Fig. 4:**
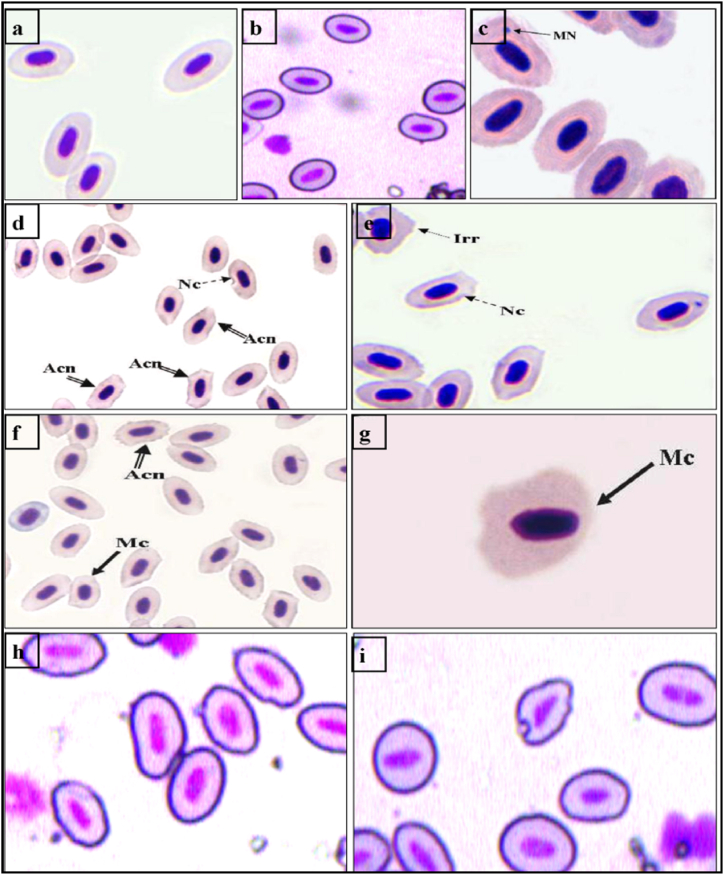
Erythrocyte cellular abnormalities of *C. carpio* treated with DM, silymarin alone and in combination, Giemsa stained (a,b) normal erythrocytes. (c-g-DM treated) (c) micronuclei (MN) (d) irregular erythrocytes (e) acanthocytes (f) notched erythrocytes (g) microcyte. (h,i) (silymarin + DM) Abbreviations: MN-Micronuclei, Nc-Notched, Acn-Acanthocyte, Mc-Microcyte, Irr-Irregular shaped erythrocytes.

**Fig. 5:**
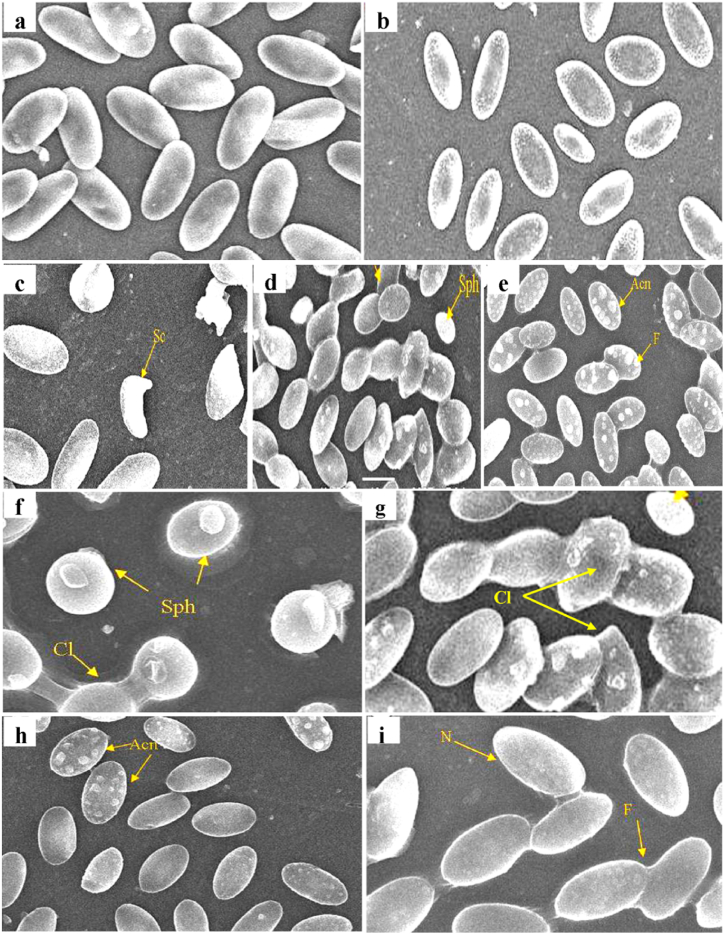
Scanning electron micrographs (SEM) of peripheral erythrocytes of *C. carpio* (a–b) control and (c–g) Exposed to DM. (h,i). Exposed to DM and treated with silymarin silymarin-supplemented diet for 30 days. Abbreviations: Acn–Acanthocyte, Cl–Clumping, Sc- Sickle-shaped cells, Sp–Spherocytes, F-Fusion of cells. N- normal cell.

In the current investigation, MN frequency in control and silymarin-treated groups did not reveal any significant change, whereas a significant (p < 0.05) increase in the MN frequency (36.15%) was registered after a 30-day exposure period to DM (Fig. 3, Fig. 4). In addition to micronucleus formation, an increase in cellular abnormalities such as irregular and notched erythrocytes, acanthocytes and microcytes, were also observed (Fig. 3B). However, fish co-treated with DM and silymarin exhibited a significant decrease (43.88%) in per cent frequency of these abnormalities compared to DM alone treated groups (see Fig. 5).

### 3.4 Ultrastructural studies

In this context, SEM studies revealed normal elliptical-shaped erythrocytes with smooth surfaces and centrally located oblong nuclei in *C. carpio*. Exposure to DM for 30 days caused marked changes in the morphology of the erythrocytes exhibiting a swollen spherical shape (spherocytosis), with their membrane depicting increased porosity. Discocytes (disc-like) and bursting erythrocytes with cytoplasmic content oozing out were among the common observations of the pesticide-treated fish (Fig. 5A–I).

However, erythrocytes of fish co-exposed to DM and silymarin exhibited slight changes in their morphology, including some irregularly shaped cells and the formation of the chain by 2–3 erythrocytes, but the severity of these alterations was very low compared to DM-exposed fish.

## 4 Discussion

Biochemical alterations are often the foremost observable and quantifiable signs of an animal's health response to environmental changes [13,56,73,74]. In this context, haematological parameters, in particular, stand out as valuable indicators for gaining insights into the toxicological consequences [[75], [76], [77], [78]].

In the present study, alteration of blood urea nitrogen (BUN), creatinine, glucose, cholesterol, and total protein levels have been evaluated in DM-exposed fish, revealing an increase in BUN and creatinine blood levels. Serum levels of urea and creatinine, serving as indicators of kidney function, were assessed. Increasing levels of these biomarkers have been found to be indicative of nephrotoxicity following DM exposure [79]. In this context, stress-induced renal dysfunction with an excessive bodily breakdown of proteins might be responsible for the elevation of urea level. Similarly, an increase in blood urea was also observed in the walking catfish (*Clarius batrachus*)*,* due to toxicity of atrazine, mancozeb, chlorpyrifos, lambda-cyhalothrin [80], and a mixture of organophosphate pesticides in the golden mahseer (*Tor putitora*) [81]. Moreover, an elevated level of creatinine and urea reflects the inability of the kidneys to completely filter out these waste products from the blood and their subsequent excretion in the urine, possibly due to cellular damage after pesticide exposure [49]. In silymarin along with DM-exposed fish, results showed a reduction in BUN and creatinine blood levels.

Moreover, results from the present study showed a decrease in protein levels. A notable decrease in overall proteins indicates a diminished capability of the system to counteract free radicals and oxidative stress. Consequently, a reduction in total protein levels may heighten the vulnerability to oxidative damage in cells and tissues, potentially contributing to a range of health issues. A decrease in protein levels has also been reported in fish exposed to DM [9] and imidacloprid [55,82]. These findings are in agreement with the results from the present study, suggesting protein degradation or biochemical transformation of protein into other nitrogenous products.

The rise in serum glucose levels induced by stress has been extensively documented in previous studies [83,84], as the measurement of glucose levels emerges as a valuable and reliable metric, serving as a key indicator that reflects the intricate dynamics of an organism's physiological state. Indeed, glucose stands as a pivotal energy source essential for fish's vitality. It plays a crucial role in maintaining optimal blood sugar levels, a balance that is intricately regulated by a multifaceted interplay of various factors such as hormones, nutritional intake, and ambient temperature. This delicate equilibrium is vital for ensuring the overall health and well-being of the organism. In the present study, serum glucose levels were revealed to have been increased in DM-exposed fish. This elevation in glucose levels may point to a disturbance in carbohydrate metabolism, potentially stemming from heightened hepatic glycogen degradation. Moreover, it is plausible that the structural alterations in the liver, induced by pesticide exposure, may have impeded the liver's capacity to store cholesterol. Dietary administration of silymarin was shown to be able to lead to a decrease the glucose levels, demonstrating silymarin's protective role against DM-induced toxicity.

The present study also investigated serum ALT, AST, and ALP level alterations. In particular, DM-exposed fish showed an increase in these enzyme levels. In this context, the elevated serum levels of AST and ALT enzyme levels in the liver could be ascribed to their release from the liver cytosol into the bloodstream, suggesting a potential compromise in the integrity and function of the hepatic membrane structure. Furthermore, the heightened activity of these transaminases suggests an intensified transamination process, likely initiated to meet the increased energy demand during stress induced by pyrethroids [85]. Hepatocyte damage may also result in alteration in ALT activity, as the enzyme is mainly localized in the cell membrane. Furthermore, as reported in the results of the present study, increased ALP activity in fish subjected to DM exposure may be indicative of potential liver damage or dysfunction. Indeed, elevated levels of ALP activity in the fish bloodstream could serve as a warning sign for liver injury or cholestasis, involving the obstruction of bile flow [51,86]. Moreover, combined treatment of DM with silymarin resulted in a gradual recovery of altered ALP activity. In this context, the modulatory effect of silymarin on other fish species has been documented. A decline in ALP activity was reported in striped catfish (*Pangasianodon hypophthalmus)* fed with silymarin supplemented diet [28], zebra cichlid (*Cichlasoma nigrofasciatum*) exposed to malathion [5], and rainbow trout (*Oncorhynchus mykiss*) specimens exposed to diazinon [29].

The hepatoprotective effects observed might be linked to the characteristics of silymarin. Indeed, it has demonstrated a significant ability to restore hepatic enzyme activities through its antioxidant scavenging actions against free radicals and metal ions. This, in turn, aids in inhibiting lipid peroxidation and safeguarding membrane permeability properties. These actions collectively work to counteract liver damage [87].

Haematological parameters serve as highly effective indicators, offering valuable insights into fish health and enabling a comprehensive understanding of their homeostasis. This understanding, in turn, can significantly aid in the monitoring of pathophysiological changes related to factors such as nutrition, water quality, or disease, making it a pivotal tool in biomonitoring efforts [88,89]. Findings showed decreased TEC and Hb levels. These results might indicate an anaemic condition caused by inhibited erythropoiesis, haemosynthesis, and amplification of RBC destruction in the hematopoietic organ [90]. Further lysing of erythrocytes due to toxicant stress may also lead to a reduction in haemoglobin and haematocrit values in the fish [91]. In contrast, TLC showed an increase after fish DM exposure. Leukocytes play a crucial role in regulating immunological function, and alterations in TLC may suggest a decline in the fish's nonspecific immunity, thus a consequence of toxicant-related activation of the immune system. This rise in this study could be attributed to a broad immune response, indicative of a protective reaction to DM-induced stress. The stimulation of lymphopoiesis and/or heightened release of lymphocytes from lymphomyeloid tissue under toxic stress may lead to an increase in TLC numbers [92]. Similarly, an alteration in TLC has been reported in *C. carpio* after exposure to chlorpyrifos [93]. In contrast, it was observed that the results of the present study, corroborate the findings of Mukherjee et al. [94].

Moreover, the micronucleus assay (MN) has been employed in order to assess DNA damage due to DM exposure. In this context, it is widely acknowledged as the most sensitive and dependable method for quantifying the genotoxicity derived from xenobiotic compounds [10]. Fish respond to toxic agents similar to higher vertebrates and can allow the assessment of substances that are potentially hazardous to humans. The MN test, due to its potentiality to be applied in any proliferating cell population regardless of the karyotype of the species used, has been successfully applied in fish to evaluate the genotoxic activity of xenobiotic agents and complex environmental mixtures in the laboratory as well as in field studies [95]. The increase in the percentage of erythrocyte structural anomalies along with micronuclei could be related to caspase-induced increased activity of DNAase, resulting in cleavage of cytoskeletal proteins and mitochondrial damage. Similar effects were observed in Nile tilapia (*Oreochromis niloticus)* specimens treated with methomyl-based pesticide and co-supplemented with brown seaweed [96] and in African catfish (*Pangasius sutchi)* specimens exposed to cadmium and co-supplemented with *Morinda tinctoria* leaf extract [97].

Blood cells play a fundamental role in the physiology of fish respiration and stand for a remarkable model to explore xenobiotic-induced damage to cellular compartments [98]. Thus, any morphological alteration will impair the mechanism of gaseous exchange. Therefore, the morphology of fish erythrocytes is one of the most sensitive indicators of the toxic impact of various environmental factors. Fish erythrocytes have the shape of biconvex discs through which O_2_ and CO_2_ rapidly diffuse to and from the interior of the cell. Hence, any morphological alteration will rapidly impair the gaseous exchange thereby resulting in hypoxaemia, hypercapnia, blood acidosis, etc. Toxic substances taken up from the water enter the blood and therefore, blood cells are among the first target of toxicity, immediately after the gill epithelium. Additionally, blood may be considered as a target and carrier of chemicals since the lipid moiety of the erythrocyte membrane is likely a site of interaction. For this reason, ultrastructure studies of erythrocytes have been performed. In this context, deformed erythrocyte cytoskeleton and swelling could be attributed to interaction with lipid moiety of the erythrocytic membrane [99], whereas, the formation of spherocytes could be due to mutant β – spectrin gene causing haemolysis [100]. Additionally, the development of acanthocytes could be correlated with defects in RBC lipid or protein composition [101]. The results of the present study confirm with findings on the grass carp (*Ctenopharyngodon Idella)* exposed to fenvalerate [102] and in the freshwater fish, (*Channa punctata*) exposed to Naphthalene-2-sulfonate [103]. In the present study, the erythrocyte membrane was most affected by DM exposure indicating increased membrane porosity and altered shape which in turn, was due to increased lipid peroxidation induced by this chemical. The alterations encountered lead to significant alterations in their structural conformation which may compromise effective blood flow, oxygen uptake and release.

Phytotherapeutics provides effective and sustainable methods for mitigating contaminant-based toxicity. Phytochemicals are reported to favour anti-stress, immunostimulation and anti-pathogen properties in fish [24,104]. Plant extracts have been reported to have antioxidant activities owing to the presence of flavonoids, alkaloids, terpenoids, saponins, phenolics, tannins, steroids and essential oils, and have been employed in aquaculture for growth promotion, anti-stress and anti-pathogen properties [105].

Supplementation of Silymarin extract with deltamethrin within the present study demonstrated effective free radical scavenging activity evinced by a decrease in induction of micronuclei, ultrastructural deformities and biochemical profile of the fish. The study validated the efficacy of silymarin as an excellent modulator of deltamethrin-induced toxicity.

## 5 Conclusion

The research findings underscore the remarkable sensitivity of *C. carpio* to DM, even at relatively low concentrations. Notably, the pesticide, induced both biochemical and haematological changes in the fish, along with DNA damage, which, if left unaddressed, could potentially result in severe ecological repercussions. Furthermore, the study provides compelling evidence supporting the ameliorative potential of silymarin as an antioxidant in mitigating DM-induced toxicity. The concurrent administration of silymarin effectively normalized elevated levels of AST, ALT, ALP, glucose, protein, and cholesterol, while also restoring various haematological parameters. A notable reduction in the number of micronuclei was also observed, reinforcing the protective role of silymarin. In light of these findings, it is strongly recommended to consider the utilization of silymarin as a protective measure to safeguard *C. carpio* as well as other fish species, as reported in the literature, from the adverse effects of DM within natural ponds and aquaculture systems. The potential beneficial impact of natural treatments, and in particular, of silymarin, requires more attention from the scientific world, as it can be a protective, enhancing, effective, and sustainable alternative to pharmaceutical treatments. For this reason, further studies in support of this need to be conducted.

## Ethical approval

Institutional Animal Ethical Committee, Panjab University, Chandigarh (PU/IAEC/S/14/151).

## Data availability statement

Data will be made available upon a reasonable request.

## CRediT authorship contribution statement

**Rajinder Jindal:** Writing – original draft, Visualization, Validation, Supervision, Software, Resources, Methodology, Investigation, Funding acquisition, Formal analysis, Data curation, Conceptualization. **Ritu Sharma:** Writing – original draft, Visualization, Validation, Supervision, Software, Resources, Methodology, Investigation, Funding acquisition, Formal analysis, Data curation, Conceptualization. **Parminder Kaur:** Writing – original draft, Resources, Methodology, Investigation, Conceptualization. **Sukhmani Kaur:** Software, Methodology, Investigation, Formal analysis, Conceptualization. **Cristiana Roberta Multisanti:** Writing – review & editing, Writing – original draft, Visualization, Validation. **Caterina Faggio:** Writing – review & editing, Supervision, Project administration.

## Declaration of competing interest

The authors declare that they have no known competing financial interests or personal relationships that could have appeared to influence the work reported in this paper.

## References

[1] Aliko V., Multisanti C.R., Turani B., Faggio C. (2022). Get rid of marine pollution: bioremediation an innovative, attractive, and successful cleaning strategy. Sustainability.

[2] Burgos-Aceves M.A., Abo-Al-Ela H.G., Faggio C. (2021). Impact of phthalates and bisphenols plasticizers on haemocyte immune function of aquatic invertebrates: a review on physiological, biochemical, and genomic aspects. J. Hazard Mater..

[3] Hamed H.S., Ismal S.M., Faggio C. (2021). Effect of allicin on antioxidant defense system, and immune response after carbofuran exposure in Nile tilapia. Oreochromis niloticus. Comp. Biochem. Physiol. C Toxicol. Pharmacol..

[4] Impellitteri F., Multisanti C.R., Rusanova P., Piccione G., Falco F., Faggio C. (2023). Exploring the impact of contaminants of emerging concern on fish and invertebrates physiology in the Mediterranean Sea. Biology.

[5] Forouhar Vajargah M., Imanpoor M.R., Shabani A., Hedayati A., Faggio C. (2019). Effect of long‐term exposure of silver nanoparticles on growth indices, hematological and biochemical parameters and gonad histology of male goldfish (Carassius auratus gibelio). Microsc. Res. Tech..

[6] Stara A., Pagano M., Albano M., Savoca S., Di Bella G., Albergamo A., Faggio C. (2021). Effects of long-term exposure of *Mytilus galloprovincialis* to thiacloprid: a multibiomarker approach. Environ. Pollut..

[7] Sula E., Aliko V., Barceló D., Faggio C. (2020). Combined effects of moderate hypoxia, pesticides and PCBs upon crucian carp fish, *Carassius carassius*, from a freshwater lake-in situ ecophysiological approach. Aquat. Toxicol..

[8] Ranatunga M., Kellar C., Pettigrove V. (2023). Toxicological impacts of synthetic pyrethroids on non-target aquatic organisms: a review. Environ. Adv..

[9] Ullah S., Li Z., Zuberi A., Arifeen M.Z.U., Baig M.M.F.A. (2019). Biomarkers of pyrethroid toxicity in fish. Environ. Chem. Lett..

[10] Sharma R., Jindal R. (2022). In vivo genotoxic effects of commercial grade cypermethrin on fish peripheral erythrocytes. Environ. Mol. Mutagen..

[11] Saha S., Chukwuka A.V., Mukherjee D., Patnaik L., Nayak S., Dhara K., Faggio C. (2021). Chronic effects of Diazinon® exposures using integrated biomarker responses in freshwater walking catfish, Clarias batrachus. Appl. Sci..

[12] Sharma S., Dar O.I., Singh K., Kaur A., Faggio C. (2021). Triclosan elicited biochemical and transcriptomic alterations in *Labeo rohita* larvae. Environ. Toxicol. Pharmacol..

[13] Sharma S., Iqbal Dar O., Andotra M., Sharma S., Kaur A., Faggio C. (2021). Environmentally relevant concentrations of Triclosan induce cyto-genotoxicity and biochemical alterations in the hatchlings of *Labeo rohita*. Appl. Sci..

[14] Sharma R., Jindal R., Faggio C. (2021). *Cassia fistula* ameliorates chronic toxicity of cypermethrin in *Catla catla*. Comp. Biochem. Physiol. C Toxicol. Pharmacol..

[15] Dar O.I., Aslam R., Pan D., Sharma S., Andotra M., Kaur A., Faggio C. (2022). Source, bioaccumulation, degradability and toxicity of triclosan in aquatic environments: a review. Environ. Technol. Innov..

[16] Ramya S., Barathinivas A., Jayakumararaj R., Pothiraj C., Ali D., Piccione G., Multisanti C.R., Faggio C. (2023). Ecotoxicological insights: effects of pesticides on ionic metabolism regulation in freshwater catfish, *Mystus keletius*. Aquat. Toxicol. (N. Y.).

[17] Plhalova L., Blahova J., Divisova L., Enevova V., Casuscelli di Tocco F., Faggio C., Svobodova Z. (2018). The effects of subchronic exposure to NeemAzal T/S on zebrafish (*Danio rerio*). Chem. Ecol..

[18] Yalsuyi A.M., Vajargah M.F., Hajimoradloo A., Galangash M.M., Prokić M.D., Faggio C. (2021). Evaluation of behavioral changes and tissue damages in common carp (*Cyprinus carpio*) after exposure to the herbicide glyphosate. Vet. Sci..

[19] Ecobichon D.J. (1991). The Basic Science of Poisons.

[20] Dogan D., Can C. (2011). Hematological, biochemical, and behavioral responses of Oncorhynchus mykiss to dimethoate. Fish Physiol. Biochem..

[21] Ahmadniaye Motlagh H., Horie Y., Rashid H., Banaee M., Multisanti C.R., Faggio C. (2023). Unveiling the effects of Fennel (*Foeniculum vulgare*) seed essential oil as a diet supplement on the biochemical parameters and reproductive function in Female common carps (*Cyprinus carpio*). Water.

[22] Beltrán J.M.G., Silvera D.G., Ruiz C.E., Campo V., Chupani L., Faggio C., Esteban M.Á. (2020). Effects of dietary Origanum vulgare on gilthead seabream (*Sparus aurata* L.) immune and antioxidant status. Fish Shellfish Immunol..

[23] Iswarya A., Vaseeharan B., Anjugam M., Gobi N., Divya M., Faggio C. (2018). β-1, 3 glucan binding protein based selenium nanowire enhances the immune status of *Cyprinus carpio* and protection against *Aeromonas hydrophila* infection. Fish Shellfish Immunol..

[24] Rashidian G., Kajbaf K., Prokić M.D., Faggio C. (2020). Extract of common mallow (Malvae sylvestris) enhances growth, immunity, and resistance of rainbow trout (*Oncorhynchus mykiss*) fingerlings against *Yersinia ruckeri* infection. Fish Shellfish Immunol..

[25] Rashidian G., Shahin K., Elshopakey G.E., Mahboub H.H., Fahim A., Elabd H., Faggio C. (2022). The dietary effects of nutmeg (*Myristica fragrans*) extract on growth, hematological parameters, immunity, antioxidant status, and disease resistance of common carp (*Cyprinus carpio*) against Aeromonas hydrophila. J. Mar. Sci. Eng..

[26] Rashidian G., Lazado C.C., Mahboub H.H., Mohammadi-Aloucheh R., Prokić M.D., Nada H.S., Faggio C. (2021). Chemically and green synthesized ZnO nanoparticles alter key immunological molecules in common carp (*Cyprinus carpio*) skin mucus. Int. J. Mol. Sci..

[27] Vazirzadeh A., Marhamati A., Rabiee R., Faggio C. (2020). Immunomodulation, antioxidant enhancement and immune genes up-regulation in rainbow trout (*Oncorhynchus mykiss*) fed on seaweeds included diets. Fish Shellfish Immunol..

[28] Abdel-Latif H.M., Shukry M., Noreldin A.E., Ahmed H.A., El-Bahrawy A., Ghetas H.A., Khalifa E. (2023). Milk thistle (*Silybum marianum*) extract improves growth, immunity, serum biochemical indices, antioxidant state, hepatic histoarchitecture, and intestinal histomorphometry of striped catfish, *Pangasianodon hypophthalmus*. Aquaculture.

[29] Banaee M., Impellitteri F., Multisanti C.R., Sureda A., Arfuso F., Piccione G., Faggio C. (2023). Evaluating silymarin extract as a potent antioxidant supplement in diazinon-exposed rainbow trout: oxidative stress and biochemical parameter analysis. Toxics.

[30] Škottová N., Krečman V., Šimánek V. (1999). Activities of silymarin and its flavonolignans upon low density lipoprotein oxidizability in vitro. Phytother Res.: An International Journal Devoted to Pharmacological and Toxicological Evaluation of Natural Product Derivatives.

[31] Pradhan S.C., Girish C. (2006). Hepatoprotective herbal drug, silymarin from experimental pharmacology to clinical medicine. Indian J. Med. Res..

[32] Ahmadi K., Banaee M., Vosoghei A.R., Mirvaghefei A.R., Ataeimehr B. (2012). Evaluation of the immunomodulatory effects of silymarin extract (*Silybum marianum*) on some immune parameters of rainbow trout, *Oncorhynchus mykiss* (Actinopterygii: salmoniformes: Salmonidae). Acta Ichthyol. Piscatoria.

[33] Banaee M., Sureda A., Mirvaghefi A.R., Rafei G.R. (2011). Effects of long-term silymarin oral supplementation on the blood biochemical profile of rainbow trout (*Oncorhynchus mykiss*). Fish Physiol. Biochem..

[34] Shiau R.J., Shih P.C., Wen Y.D. (2011).

[35] Shaker E., Mahmoud H., Mnaa S. (2010). Silymarin, the antioxidant component and Silybum marianum extracts prevent liver damage. Food Chem. Toxicol..

[36] El-Shitany N.A., El-Haggar S., El-Desoky K. (2008). Silymarin prevents adriamycin-induced cardiotoxicity and nephrotoxicity in rats. Food Chem. Toxicol..

[37] Soto C., Pérez J., García V., Uría E., Vadillo M., Raya L. (2010). Effect of silymarin on kidneys of rats suffering from alloxan-induced diabetes mellitus. Phytomedicine.

[38] Toklu H.Z., Akbay T.T., Velioglu-Ogunc A., Ercan F., Gedik N., Keyer-Uysal M., Sener G. (2008). Silymarin, the antioxidant component of *Silybum marianum*, prevents sepsis-induced acute lung and brain injury. J. Surg. Res..

[39] Li L., Sun H.Y., Liu W., Zhao H.Y., Shao M.L. (2017). Silymarin protects against acrylamide-induced neurotoxicity via Nrf2 signalling in PC12 cells. Food Chem. Toxicol..

[40] Simánek V., Kottová N., Bartek J., Psotová J., Kosina P., Balejová L., Ulrichová J. (2000). Extract from *Silybum marianum* as a nutraceutical: a double-blind placebo-controlled study in healthy young men. Czech J. Food Sci..

[41] Arvanag F.M., Bayrami A., Habibi-Yangjeh A., Pouran S.R. (2019). A comprehensive study on antidiabetic and antibacterial activities of ZnO nanoparticles biosynthesized using *Silybum marianum* L seed extract. Mater. Sci. Eng. C.

[42] Wang J., Zhou H., Wang X., Mai K., He G. (2019). Effects of silymarin on growth performance, antioxidant capacity and immune response in turbot (Scophthalmus maximus L.). J. World Aquacult. Soc..

[43] Xiao P., Ji H., Ye Y., Zhang B., Chen Y., Tian J., Du Z. (2017). Dietary silymarin supplementation promotes growth performance and improves lipid metabolism and health status in grass carp (*Ctenopharyngodon idellus*) fed diets with elevated lipid levels. Fish Physiol. Biochem..

[44] Multisanti C.R., Merola C., Perugini M., Aliko V., Faggio C. (2022). Sentinel species selection for monitoring microplastic pollution: a review on one health approach. Ecol. Indicat..

[45] Darabi H., Baradaran A., Ebrahimpour K. (2022). Subacute toxic effects of polyvinyl chloride microplastics (PVC-MPs) in juvenile common carp, *Cyprinus carpio* (Pisces: cyprinidae). Casp. J. Environ. Sci..

[46] Yalsuyi A.M., Vajargah M.F., Hajimoradloo A., Galangash M.M., Prokić M.D., Faggio C. (2021). Evaluation of behavioral changes and tissue damages in common carp (*Cyprinus carpio*) after exposure to the herbicide glyphosate. Vet. Sci..

[47] Mahboub H.H., Faggio C., Hendam B.M., Algharib S.A., Alkafafy M., Hashem M.A., Rahman A.N.A. (2022). Immune-antioxidant trait, *Aeromonas veronii* resistance, growth, intestinal architecture, and splenic cytokines expression of *Cyprinus carpio* fed *Prunus armeniaca* kernel-enriched diets. Fish Shellfish Immunol..

[48] Barathinivas A., Ramya S., Neethirajan K., Jayakumararaj R., Pothiraj C., Balaji P., Faggio C. (2022). Ecotoxicological effects of pesticides on hematological parameters and oxidative enzymes in freshwater Catfish, *Mystus keletius*. Sustainability.

[49] El Hajam M., Plavan G.I., Kandri N.I., Dumitru G., Nicoara M.N., Zerouale A., Faggio C. (2020). Evaluation of softwood and hardwood sawmill wastes impact on the common carp" *Cyprinus carpio*" and its aquatic environment: an oxidative stress study. Environ. Toxicol. Pharmacol..

[50] Hatami M., Banaee M., Haghi B.N. (2019). Sub-lethal toxicity of chlorpyrifos alone and in combination with polyethylene glycol to common carp (*Cyprinus carpio*). Chemosphere.

[51] Banaee M., Badr A.A., Multisanti C.R., Haghi B.N., Faggio C. (2023). The toxicity effects of the individual and combined exposure of methyl tert-butyl ether (MTBE) and tire rubber powder (RP) on Nile tilapia fish (*Oreochromis niloticus*). Comp. Biochem. Physiol. C Toxicol. Pharmacol..

[52] Zeidi A., Sayadi M.H., Rezaei M.R., Banaee M., Gholamhosseini A., Pastorino P., Multisanti C.R., Faggio C. (2023). Single and combined effects of Cuso4 and polyethylene microplastics on biochemical endpoints and physiological impacts on the narrow-clawed crayfish pontastacus Leptodactylus. Chemosphere.

[53] Farag M.R., Alagawany M. (2018). Erythrocytes as a biological model for screening of xenobiotics toxicity. Chem. Biol. Interact..

[54] Mohanty J.G., Nagababu E., Rifkind J.M. (2014). Red blood cell oxidative stress impairs oxygen delivery and induces red blood cell aging. Frontiers physiol..

[55] Alvim T.T., dos Reis Martinez C.B. (2019). Genotoxic and oxidative damage in the freshwater teleost Prochilodus lineatus exposed to the insecticides lambda-cyhalothrin and imidacloprid alone and in combination. Mutat. Res. Genet. Toxicol. Environ. Mutagen.

[56] Burgos-Aceves M.A., Lionetti L., Faggio C. (2019). Multidisciplinary haematology as prognostic device in environmental and xenobiotic stress-induced response in fish. Sci. Total Environ..

[57] Saha S., Dhara K., Chukwuka A.V., Pal P., Saha N.C., Faggio C. (2023). Sub-lethal acute effects of environmental concentrations of inorganic mercury on hematological and biochemical parameters in walking catfish, *Clarias batrachus*. Comp. Biochem. Physiol., Part C: Toxicol. Pharmacol..

[58] Chen H., Luo D. (2023). Application of haematology parameters for health management in fish farms. Rev. Aquacult..

[59] APHA (2012).

[60] ASTM International (2003).

[61] Finney D.J. (1980).

[62] Zahra S.A., Liu W., Si S. (2023). How digital technology promotes entrepreneurship in ecosystems. Technovation.

[63] Henry R.J., Cannon D.C., Winkelman W. (1974).

[64] Chaney A.L., Marbach E.P. (1962). Modified reagents for determination of urea and ammonia. Clin. Chem..

[65] Slot C. (1965). Plasma creatinine determination a new and specific Jaffe reaction method. Scand. J. Clin. Lab. Invest..

[66] Trinder P. (1969). Determination of glucose using glucose oxidase with an alternative oxygen acceptor. Ann. Clin. Biochem..

[67] Lowry O.H., Rosebrough N.J., Farr A.L., Randall R.J. (1951). Protein measurement with the Folin phenol reagent. J. Biol. Chem..

[68] Roeschlau P., Bernt E., Gruber W.J. (1974). Enzymatic determination of total cholesterol in serum. J. Clin. Chem. Clin. Biochem..

[69] Conceição M.B., Gomes J.A.A., Bucker A., Carvalho M.S. (2012). Micronucleus test and comet assay in erythrocytes of the Amazonian electric fish *Apteronotus bonapartii* exposed to benzene. Journal of the Brazilian Society of Ecotoxicology.

[70] Anbumani S., Mohankumar M.N. (2012). Gamma radiation-induced micronuclei and erythrocyte cellular abnormalities in the fish *Catla catla*. Aquat. Toxicol. (N. Y.).

[71] Simpson L.O. (1989). Blood from healthy animals and humans contains nondiscocytic erythrocytes. Br. J. Haematol..

[72] Witeska M., Kondera E., Ługowska K., Bojarski B. (2022). Hematological methods in fish–Not only for beginners. Aquaculture.

[73] Rashidian G., Mohammadi-Aloucheh R., Hosseinzadeh-Otaghvari F., Chupani L., Stejskal V., Samadikhah H., Multisanti C.R., Faggio C. (2023). Long-term exposure to small-sized silica nanoparticles (SiO2-NPs) induces oxidative stress and impairs reproductive performance in adult zebrafish (*Danio rerio*). Comp. Biochem. Physiol. C Toxicol. Pharmacol..

[74] Shahjahan M., Taslima K., Rahman M.S., Al-Emran M., Alam S.I., Faggio C. (2022). Effects of heavy metals on fish physiology–a review. Chemosphere.

[75] Ahmed I., Reshi Q.M., Fazio F. (2020). The influence of the endogenous and exogenous factors on hematological parameters in different fish species: a review. Aquacult. Int..

[76] Banaee M., Faraji J., Amini M., Multisanti C.R., Faggio C. (2023). Rainbow trout (*Oncorhynchus mykiss*) physiological response to microplastics and enrofloxacin: novel pathways to investigate microplastic synergistic effects on pharmaceuticals. Aquat. Toxicol..

[77] Multisanti C.R., Riolo K., Impellitteri F., Chebbi I., Faggio C., Giannetto A. (2023). Short-term in vitro exposure of *Pinctada imbricata*'s haemocytes to quaternium-15: exploring physiological and cellular responses. Environ. Toxicol. Pharmacol..

[78] Shahjahan M., Taslima K., Rahman M.S., Al-Emran M., Alam S.I., Faggio C. (2022). Effects of heavy metals on fish physiology–a review. Chemosphere.

[79] Harabawy A.S., Ibrahim A.T.A. (2014). Sublethal toxicity of carbofuran pesticide on the African catfish Clarias gariepinus (Burchell, 1822): hematological, biochemical and cytogenetic response. Ecotoxicol. Environ. Saf..

[80] Kanu K.C., Okoboshi A.C., Otitoloju A.A. (2023). Haematological and biochemical toxicity in freshwater fish *Clarias gariepinus* and *Oreochromis niloticus* following pulse exposure to atrazine, mancozeb, chlorpyrifos, lambda-cyhalothrin, and their combination. Comp. Biochem. Physiol., Part C: Toxicol. Pharmacol..

[81] Kunwar P.S., Sinha A.K., De Boeck G., Sapkota K. (2022). Modulations of blood biochemical parameters of golden mahseer, *Tor putitora* following exposures to single and mixed organophosphate. Comp. Biochem. Physiol. C Toxicol. Pharmacol..

[82] Ilahi I., Ullah S., Ali H., Begum R., Nawaz H., Bibi H., Sheema B.S. (2018). Effect of long term exposure to sublethal concentration of imidacloprid on some biochemical and haematological parameters of Grass carp and Goldfish. Pak. J. Pharm. Sci..

[83] Evans T.G., Kültz D. (2020). The cellular stress response in fish exposed to salinity fluctuations. J. Exp. Zool.: Ecol. Integr. Physiol.

[84] Naz S., Iqbal S., Khan R.U., Chatha A.M.M., Naz S., Ahmad M.I., Mahamood M., Javed M., Alhewairini S.S. (2023). Toxicology and Human Health.

[85] Reddy D.R.K. (2023). Toxicological effect of endocrine disrupting insecticide (DM) on enzymatical, haematological and histopathological changes in the freshwater iridescent shark, *Pangasius hypothalamus*. Environ. Toxicol. Pharmacol..

[86] Lotfi S., Esfahani M., Ranjbar A., Mehri F. (2023). Protective effect of flaxseed oil the on diazinon-induced hepatotoxicity in male rats. Proc. Indian Natl. Sci. Acad..

[87] Borsari M., Gabbi C., Ghelfi F., Grandi R., Saladini M., Severi S., Borella F. (2001). Silybin, a new iron-chelating agent. J. Inorg. Biochem..

[88] Vali S., Majidiyan N., Yalsuyi A.M., Vajargah M.F., Prokić M.D., Faggio C. (2022). Ecotoxicological effects of silver nanoparticles (Ag-NPs) on parturition time, survival rate, reproductive success and blood parameters of adult common molly (*Poecilia sphenops*) and their larvae. Water.

[89] Banaee M., Beitsayah A., Prokić M.D., Petrović T.G., Zeidi A., Faggio C. (2023). Effects of cadmium chloride and biofertilizer (Bacilar) on biochemical parameters of freshwater fish, Alburnus mossulensis. Comp. Biochem. Physiol., Part C: Toxicol. Pharmacol..

[90] Yonar S.M., Ural M.S., Silici S., Yonar M.E. (2014). Malathion-induced changes in the haematological profile, the immune response, and the oxidative/antioxidant status of *Cyprinus carpio*: protective role of propolis. Ecotoxicol. Environ. Saf..

[91] Saravanan M., Kumar K.P., Ramesh M. (2011). Haematological and biochemical responses of freshwater teleost fish *Cyprinus carpio* (Actinopterygii: cypriniformes) during acute and chronic sublethal exposure to lindane. Pestic. Biochem. Physiol..

[92] El-Sayed Y.S., Saad T.T., El-Bahr S.M. (2007). Acute intoxication of deltamethrin in monosex Nile tilapia, *Oreochromis niloticus* with special reference to the clinical, biochemical and haematological effects. Environ. Toxicol. Pharmacol..

[93] Ghayyur S., Tabassum S., Ahmad M.S., Akhtar N., Khan M.F. (2019). Effect of chlorpyrifos on hematological and seral biochemical components of fish *Oreochromis mossambicus*. Pakistan J. Zool..

[94] Mukherjee D., Ferreira N.G., Saha N.C. (2022). Effects of 2, 4, 6-Trichlorophenol on *Clarias batrachus:* a biomarkers approach. Environ. Sci. Pollut. Res..

[95] Oliveira C., Foresti F., Hilsdorf A.W.S. (2009). Genetics of neotropical fish: from chromosomes to populations. Fish Physiol. Biochem..

[96] Kilawati Y., Islamy R.A. (2019). The antigenotoxic activity of brown seaweed (Sargassum sp.) extract against total erythrocyte and micronuclei of *Tilapia Oreochromis* niloticus exposed by methomyl-base pesticide. J. Exp. Life Sci.

[97] DeiviArunachalam K., Kuruva J.K., Pradhoshini K.P., Musthafa M.S., Faggio C. (2021). Antioxidant and antigenotoxic potential of Morinda tinctoria Roxb. leaf extract succeeding cadmium exposure in Asian catfish, Pangasius sutchi. Comp. Biochem. Physiol. C Toxicol. Pharmacol..

[98] Sula E., Aliko V., Pagano M., Faggio C. (2020). Digital light microscopy as a tool in toxicological evaluation of fish erythrocyte morphological abnormalities. Microsc. Res. Tech..

[99] Podsiedlik M., Markowicz-Piasecka M., Sikora J. (2020). Erythrocytes as model cells for biocompatibility assessment, cytotoxicity screening of xenobiotics and drug delivery. Chem. Biol. Interact..

[100] Park J., Jeong D.C., Yoo J., Jang W., Chae H., Kim J., Kim M. (2016). Mutational characteristics of ANK1 and SPTB genes in hereditary spherocytosis. Clin. Genet..

[101] Sawhney A.K., Johal M.S. (2000). Erythrocyte alterations induced by malathion in *Channa punctatus* (Bloch). Bull. Environ. Contam. Toxicol..

[102] Saha S., Dhara K., Pal P., Saha N.C., Faggio C., Chukwuka A.V. (2023). Longer-term adverse effects of selenate exposures on hematological and serum biochemical variables in air-breathing fish *Channa punctata* (Bloch, 1973) and non-air breathing fish *Ctenopharyngodon Idella* (Cuvier, 1844): an integrated biomarker response approach. Biol. Trace Elem. Res..

[103] Mehra S., Chadha P. (2021). Naphthalene-2-sulfonate induced toxicity in blood cells of freshwater fish *Channa punctatus* using comet assay, micronucleus assay and ATIR-FTIR approach. Chemosphere.

[104] Ahmadifar E., Sheikhzadeh N., Roshanaei K., Dargahi N., Faggio C. (2019). Can dietary ginger (*Zingiber officinale*) alter biochemical and immunological parameters and gene expression related to growth, immunity and antioxidant system in zebrafish (*Danio rerio*)?. Aquacult.

[105] Citarasu T. (2010). Herbal biomedicines: a new opportunity for aquaculture industry. Aquacult. Int..

